# Secondary Prevention Medical Therapy and Outcomes in Patients With Myocardial Infarction With Non-Obstructive Coronary Artery Disease

**DOI:** 10.3389/fphar.2019.01606

**Published:** 2020-01-31

**Authors:** Pasquale Paolisso, Luca Bergamaschi, Giulia Saturi, Emanuela Concetta D'Angelo, Ilenia Magnani, Sebastiano Toniolo, Andrea Stefanizzi, Andrea Rinaldi, Lorenzo Bartoli, Francesco Angeli, Francesco Donati, Paola Rucci, Anna Vittoria Mattioli, Nevio Taglieri, Carmine Pizzi, Nazzareno Galiè

**Affiliations:** ^1^Department of Experimental, Diagnostic and Specialty Medicine-DIMES, University of Bologna, Bologna, Italy; ^2^Division of Hygiene and Biostatistics, Department of Biomedical and Neuromotor Sciences, Alma Mater Studiorum, University of Bologna, Bologna, Italy; ^3^Department of Surgical, Medical and Dental Department of Morphological Sciences Related to Transplant, Oncology and Regenerative Medicine, University of Modena and Reggio Emilia, Modena, Italy

**Keywords:** myocardial infarction with non-obstructive coronary arteries (MINOCA), secondary prevention medical therapy, prognosis, RAAS inhibitors, β-blockers, statins, dual antiplatelet therapy (DAPT)

## Abstract

**Background:**

Myocardial infarction with non-obstructive coronary arteries (MINOCA) is a heterogeneous entity with relevant long-term major cardiovascular events. Several trials have demonstrated that dual antiplatelet therapy (DAPT), β-blocker, renin-angiotensin-aldosterone system (RAAS) inhibitor and statin therapy improve the prognosis in patients with obstructive myocardial infarction (ob-MI). However, evidence on the best medical therapy for secondary prevention in MINOCA patients is lacking.

**Purpose:**

To investigate the effects of secondary prevention treatments at discharge on mid-term outcomes in MINOCA.

**Methods:**

Patients with acute myocardial infarction (MI) undergoing early coronary angiography between 2016 and 2018 were extracted from a clinical database. The diagnosis of MINOCA was made according to 2016 ESC MINOCA Position Paper criteria. Second-level diagnostic work-up including cardiac magnetic resonance was performed to exclude non-ischemic troponin elevation cause. The relationship between treatments and outcomes was evaluated by using Kaplan-Meier survival analysis and Cox regression models. All confirmed MINOCA were followed in our outpatient clinics. The primary end-points were all-cause mortality, re-hospitalization for MI and a composite outcome including all-cause mortality, hospitalization for MI and ischemic stroke (MACE).

**Results:**

Out of 1,141 AMI who underwent coronary angiography, 134 were initially diagnosed as MINOCA. Patients with MINOCA were less likely to receive secondary prevention treatments than patients with obstructive coronary artery disease (CAD) MI (respectively, 42.1% vs 81.8% for DAPT; 75.5% vs 89.6% for β-blockers; 64.7% vs 80.3% for RAAS inhibitor and 63.9% vs 83% for statins). Based on the diagnostic work-up completed during the first month after discharge, a final sample of 88 patients had confirmed MINOCA. During an average follow-up of 19.35 ± 10.65 months, all-cause mortality occurred in 11 (12.5%) patients, recurrence of MI in 4 (4.5%), and MACE in 15 (17.0%) patients. Patients treated with RAAS inhibitors and statins had a significantly longer survival. On the contrary, no increase in survival was found in patients treated with β-blockers or DAPT. Cox multivariable analysis, including all secondary prevention drugs, showed that only RAAS inhibitors were associated with reduced all cause-mortality and MACE.

**Conclusion:**

This prospective study suggests that RAAS inhibitor therapy provides mid-term beneficial effects on outcomes in MINOCA patients; in contrast, dual antiplatelet, β-blocker and statin therapy had no effects on mortality and MACE. These results should be considered preliminary and warrant confirmation from larger studies.

## Introduction

The 2018 ESC guidelines of the Fourth Universal definition of myocardial infarction (MI) differentiated myocardial infarction from myocardial injury and defined acute myocardial infarction with non-obstructive coronary arteries (MINOCA). According to the pathophysiological mechanisms, MINOCA were located between type I and type II MI: type 1 MI caused by atherosclerotic plaque disruption, and type 2 MI due to non athero-thrombosis plaque (epicardial coronary vasospasm, coronary microvascular dysfunction, coronary thromboembolism, spontaneous coronary artery dissection, supply-demand mismatch) (Thygesen et al., 2019).

The diagnosis of MINOCA includes the criteria for acute MI, no evidence of angiographic coronary obstruction (coronary stenosis <50%) and of a clinically apparent alternative diagnosis for the acute presentation (Agewall et al., 2017). In this definition, both patients with normal coronary arteries (without coronary atheromasia) and those with coronary atheromasia up to 50% are included (Rossini et al., 2013). Moreover, the third condition differentiating MINOCA is that it occurs in an ischemia setting, excluding other causes of non-ischemic troponin elevation (eg sepsis, pulmonary embolism, myocarditis).

Similarly to heart failure, MINOCA should be considered as a “working diagnosis” that requires further evaluation to investigate its underlying mechanisms, with important diagnostic and prognostic implications on secondary prevention therapy. Consequently, the term MINOCA designates a heterogeneous subgroup of patients with myocardial infarction, with the afore-mentioned characteristics, rather than a specific pathophysiological mechanism (Tamis-Holland et al., 2019).

Nowadays, the prevalence of MINOCA is estimated at 5–8%, with variations depending on the proportion of patients with MI who performed coronary angiography and on the high-sensitivity cardiac troponin (hs-cTn) assays used (Pasupathy et al., 2015).

Literature prognosis data showed that MINOCA is not a benign condition, with an overall estimated mortality around 3.5–4.5% (Pasupathy et al., 2015; Baron et al., 2016; Pizzi et al., 2016; Barr et al., 2018). This unfavorable outcome might be explained, at least in part, by the low rate of β-blockers, angiotensin converting enzyme inhibitors (ACEI), statins, and antiplatelet drugs prescription.

Moreover, most drugs used for secondary prevention in patients with myocardial infarction and obstructive coronary arteries are aimed at atherosclerotic disease prevention, that is a minor problem in MINOCA. Therefore, doubts have been raised about the use of standard therapy.

In three observational studies, ACEI/angiotensin Receptor Blockers (ARB) therapy has demonstrated a beneficial effect on outcome in patients with MINOCA (Manfrini et al., 2014; Lindahl et al., 2017; Choo et al., 2019). However, these studies have some potential biases, such as the diagnosis of MINOCA that includes other conditions without acute ischemia, and the long period of enrolment in which the diagnostic criteria of MI were refined. Therefore, we investigated prospectively the association between antiplatelet therapy, renin-angiotensin-aldosterone system (RAAS) inhibitors, β-blockers and statins and mid-term cardiovascular events in MINOCA patients.

## Materials and Methods

### Study Population

Out of 1,523 consecutive patients with acute myocardial infarction admitted between January 2016 and December 2018 to Bologna University Hospital (Policlinico Sant'Orsola-Malpighi), 1,141 underwent coronary angiography (CAG) within the first 48 h of the index hospitalization. According to the extent of stenosis, patients were classified into three groups: a) obstructive CAD (ob-CAD), defined as a stenosis ≥50% of the lumen diameter in at least one coronary artery, b) non-obstructive CAD (non ob-CAD), defined as a stenosis 1–49% of the coronary artery diameter, and (c) normal coronary arteries (NCA), defined as no angiographic visible stenosis or atheromasia. The MINOCA diagnosis was performed according to 2016 ESC MINOCA Position Paper criteria (Agewall et al., 2017).

**Figure 1:**
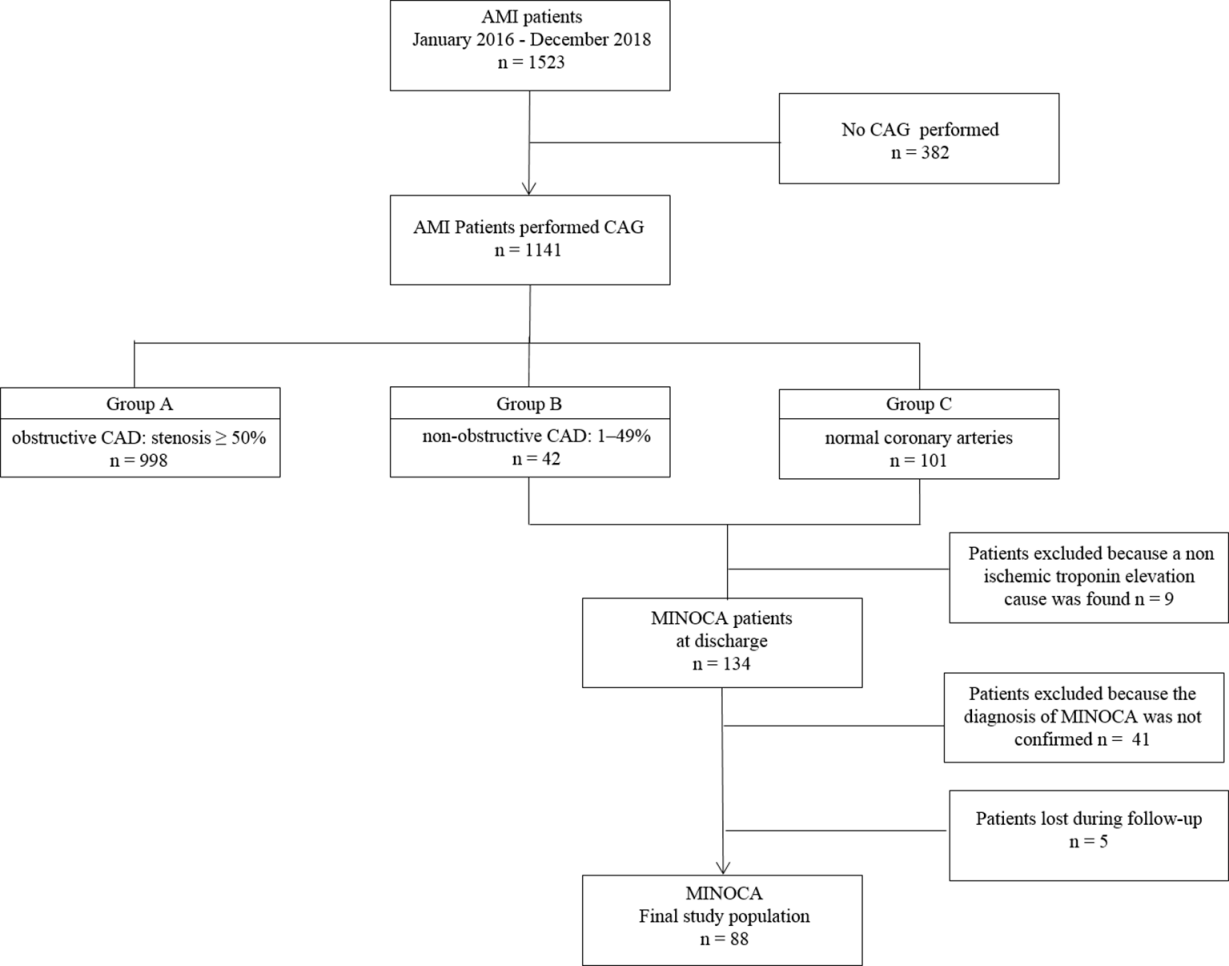
Study flow-chart. AMI: acute myocardial infarction; CAD, coronary artery disease; MINOCA, myocardial infarction with nonobstructive coronary arteries.

We excluded patients who did not perform coronary angiography or performed it after 48 h of hospital admission and patients with previous coronary revascularization (**Figure 1**).

### Patient Characteristics

For each patient, demographic and baseline clinical data were collected, including sex, age, height, weight, body mass index (BMI), cardiovascular risk factors, family history of cardiovascular disease, and first admission diagnosis. We also collected information on major epicardial coronary arteries obstruction, based on visual assessment during acute CAG performed by an expert interventional cardiologist. Discharge medications were also recorded (antiplatelet, β-blockers, RAAS inhibitors and statins). Antiplatelet therapy was prescribed according to the current guideline recommendations (Valgimigli et al., 2018).

A standard 12*-*lead ECG was performed and laboratory testing was carried out in all patients, including hemoglobin and creatinine. The glomerular filtration rate was calculated using CKD-EPI formula.

All patients underwent 2D-echocardiogram and the left ventricular ejection fraction (LVEF) was calculated, according to European recommendations (Lang et al., 2015).

During hospitalization or within 1-month of discharge, patients underwent contrast cardiac magnetic resonance (CMR) and/or pulmonary and vascular computed tomography in order to exclude non-ischemic troponin elevation causes (Tamis-Holland et al., 2019).Moreover, patients who did not complete the diagnostic work-up and those who died during the index hospitalization were excluded from the analysis.

### Ethics

Our data are collected as part of an approved protocol regarding the observational study “Acute Myocardial Infarction; Prognostic and Therapeutic Evaluation” (ClinicalTrials.gov Identifier: NCT03883711). All patients were treated in accordance with the Declaration of Helsinki; all of them were informed about their participation in the registry and provided informed consent for the anonymous publication of scientific data.

### Primary Outcomes

The primary outcomes were all-cause mortality, re-hospitalization for MI and a composite outcome including all-cause mortality, hospitalization for MI and ischemic stroke (MACE). Myocardial infarction was diagnosed according to current ESC guidelines; (1) stroke was defined as an ischemic cerebral infarction caused by embolic or thrombotic occlusion of a major intracranial artery.

The main purpose of this study was to compare the effects of secondary prevention treatments (antiplatelet, β-blockers, RAAS inhibitors, and statins) prescribed at discharge on mid-term prognosis.

### Mid-Term Follow-Up

Patients were followed from discharge through outpatient clinical visits or telephone interviews every 6 months until the end of follow-up. During the follow-up visit, each patient underwent ECG, echocardiogram and was clinically evaluated by an experienced cardiologist, who recorded drug compliance and the clinical end-points.

### Statistical Analyses

Patient characteristics were summarized using mean ± standard deviation for continuous variables and absolute and percentage frequencies for categorical variables. Patient subgroups were compared using χ² test or Fisher’s exact test for categorical variables and t-test or Mann-Whitney test for continuous variables, as appropriate.

Patients’ survival at 36 months was obtained using Kaplan-Meier estimates and compared between subgroups using log-rank test. Univariate and multivariable Cox proportional-hazard models were used to analyze the association of pharmacological treatments with death, re-infarction, stroke and MACE (either of the three major cardiovascular events).

## Results

Out of 143 patients with non-obstructive CAD or normal coronary arteries, 9 were excluded because a non-ischemic troponin elevation cause was found (e.g. sepsis, pulmonary embolism, myocarditis) during hospitalization. The diagnosis of MINOCA was made at discharge in 134 patients. Forty-one patients were further excluded because the diagnosis was not confirmed by second-level instrumental examination performed after discharge or for incomplete diagnostic work-up and 5 patients were not traced at follow-up. The final sample used to analyze the effects of secondary prevention treatments on outcomes consisted of 88 patients (**Figure 1**).

### MINOCA Versus Obstructive Coronary Artery Disease: Demographic and Clinical Characteristics

We first compared the characteristics of patients with MINOCA and those with obstructive CAD (ob-CAD). Patients with MINOCA were younger, more frequently female, and had less frequently hypertension, dyslipidemia and type-2 diabetes than patients with ob-CAD (**Table 1**). Moreover, MINOCA patients were less likely to have a medical history of previous MI (7.7% vs. 25% in ob-CAD) and had significantly higher LVEF and glomerular filtration rate.

**Table 1:** Baseline characteristics and drug prescription at discharge in MINOCA patients' vs obstructive CAD patients.

	MINOCA Patients (N = 134)	Obstructive CAD Patients (N = 998)	P-value
Age years, mean (SD)	65.73 (14.03)	69.13 (12.6)	**0.017**
Females, n (%)	79 (59)	304 (30.5)	**<0.001**
BMI kg/m2, mean (SD)	27.16 (4.52)	27.12 (4.88)	0.89
*Cardiovascular risk factors* Current/past smoking, n (%)	73 (54.9)	622 (63.2)	0.063
Hypertension, n (%)	82 (61.7)	759 (77)	**<0.001**
Dyslipidemia, n (%)	82 (61.7)	701 (71)	**0.028**
Type-2 diabetes, n (%)	14 (10.5)	239 (24.2)	**<0.001**
*Medical history* Previous MI, n (%)	9 (7.7)	244 (Tzanidis et al., 2001)	**<0.001**
Previous stroke, n (%)	9 (6.7)	94 (9.4)	0.31
Atrial fibrillation, n (%)	8 (Barr et al., 2018)	87 (8.8)	0.28
*Admission diagnosis* STEMI, n (%)	23 (18.9)	457 (49.8)	**<0.001**
*Laboratory parameters* GFR CKD-EPI ml/min, mean (SD)	75.09 (23.19)	70.37 (23.18)	**0.029**
Hemoglobin g/dl, mean (SD)	13.46 (1.75)	13.47 (2.15)	0.99
*Echocardiogram* LVEDV ml, mean (SD)	90.64 (25.80)	108.29 (40.30)	**0.046**
% EF, mean (SD)	55.67 (11.01)	52.02 (10.99)	**0.001**
*Medical Therapy at discharge* Single antiplatelet, n (%)	101 (75.9)	899 (94.6)	**<0.001**
ASA, n (%)	98 (73.7)	867 (89.6)	**<0.001**
P2Y12 inhibitor, n (%)	60 (45.1)	824 (85.1)	**<0.001**
Dual antiplatelet therapy, n (%)	56 (42.1)	787 (81.1)	**<0.001**
Beta-blockers, n (%)	100 (75.2)	855 (89.6)	**<0.001**
RAAS inhibitor, n (%)	86 (64.7)	779 (80.3)	**<0.001**
Statins, n (%)	85 (63.9)	792 (83)	**<0.001**

BMI, body mass index; MI, myocardial infarction; STEMI, ST elevation myocardial infarction; NSTEMI, non ST elevation myocardial infarction; GFR, glomerular filtration rate; CKD-EPI, Chronic Kidney Disease Epidemiology Collaboration; CRP, C-reactive Protein; LVEDd, left ventricular end diastolic diameter; LVEDV, left ventricular end diastolic volume; EF, ejection fraction; RAAS, renin-angiotensin-aldosterone system; SD, Standard deviation.

Chi-square test for categorical variables; Student's t-test for normally distributed continuous variables and Mann-Whitney test and for non-normally distributed continuous variables.

Overall, the diagnosis of ST-Elevation Myocardial Infarction at baseline was markedly less frequent among MINOCA vs ob-CAD patients (18.9%, versus 49.8%, respectively).

Of the 134 MINOCA patients (**Table 2**), only 22 had non-obstructive coronary stenosis, 19 patients had one-vessel, 2 had two-vessel disease and only 1 three-vessel disease. On the contrary, a higher prevalence of three-vessel and left main was found in patients with obstructive myocardial infarction. The mean percentage of coronary stenosis in MINOCA patients was 12.7%.

**Table 2:** Results of coronary angiography in MINOCA versus obstructive CAD patients.

	MINOCA (N = 134)	Obstructive CAD (N = 998)	P-value
One vessel disease, n (%)	19 (14.2%)	631 (63.2%)	<0.001
Two vessel disease, n (%)	2 (1.5%)	235 (23.5%)	<0.001
Three vessel disease, n (%)	1 (0.7%)	132 (13.2%)	<0.001
			
Mean stenosis % (SD)	12.7% (± 19.5)	85.2% (± 28.9)	<0.001
			
*Coronary vessel*			
Left main CAD, n (%)	8 (6.0%)	176 (17.6%)	0.001
LAD CAD, n (%)	18 (13.4%)	492 (49.3%)	<0.001
ID CAD, n (%)	3 (2.2%)	160 (16%)	<0.001
LCx CAD, n (%)	5 (3.7%)	207 (20.7%)	<0.001
OM CAD, n (%)	5 (3.7%)	183 (18.3%)	<0.001
RCA CAD, n (%)	9 (6.7%)	369 (37%)	<0.001

CAD, coronary artery disease; LAD, left anterior descending artery; ID, first diagonal branch; LCx, left circumflex artery; OM obtuse marginal branches; MINOCA, Myocardial infarction with non-obstructive coronary arteries; RCA, right coronary artery.

MINOCA patients were then classified into non ob-CAD and NCA patients. No significant difference in baseline clinical characteristics (**Table 3**) was found between these subgroups. Therefore, in the subsequent analyses, MINOCA patients were considered as a whole.

**Table 3:** Baseline characteristics and drug prescription at discharge in MINOCA sub-groups.

	Non-obstructive CAD Patients (N = 22)	Normal coronary arteries Patients (N = 66)	P-value
Age years, mean (SD)	69.32 (10.45)	66.63 (14.17)	0.59
Gender female, n (%)	13 (59.1)	42 (63.6)	0.70
BMI kg/m2, mean (SD)	25.68 (4.73)	25.65 (4.05)	0.98
*Cardiovascular risk factors* Current/past smoking, n (%)	12 (54.5)	33 (50.8)	0.76
Hypertension, n (%)	14 (63.6)	46 (70.8)	0.53
Dyslipidemia, n (%)	15 (68.2)	46 (70.8)	0.82
Type-2 diabetes, n (%)	3 (13.6)	8 (12.3)	0.87
*Medical history* Previous MI, n (%)	1 (5.3)	10 (19.2)	0.15
Previous stroke, n (%)	2 (9.1)	6 (9.1)	1.0
Atrial fibrillation, n (%)	2 (9.1)	4 (6.2)	0.64
*Admission diagnosis* STEMI, n (%)	4 (19.0)	8 (13.1)	0.51
*Laboratory parameters* GFR CKD-EPI ml/min, mean (SD)	72.62 (24.02)	73.71 (23.46)	0.76
Hemoglobin g/dl, mean (SD)	13.33 (1.94)	13.51 (1.75)	0.73
*Echocardiogram* LVEDV ml, mean (SD)	87.67 (25.24)	87.46 (25.67)	0.97
% EF, mean (SD)	57.78 (13.63)	57.27 (7.92)	0.51
*Medical Therapy at discharge* Single antiplatelet, n (%)	20 (90.9)	57 (86.4)	0.58
ASA, n (%)	19 (86.4)	56 (84.8)	0.86
P2Y12 inhibitor, n (%)	17 (77.3)	37 (56.1)	0.07
Dual antiplatelet therapy, n (%)	16 (72.7)	34 (51.5)	0.08
Beta-blockers, n (%)	20 (90.9)	57 (86.4)	0.58
RAAS inhibitor, n (%)	14 (63.6)	49 (74.2)	0.34
Statins, n (%)	18 (81.8)	48 (72.7)	0.39

BMI, body mass index; MI, myocardial infarction; STEMI, ST elevation myocardial infarction; NSTEMI, non ST elevation myocardial infarction; GFR, glomerular filtration rate; CKD-EPI, Chronic Kidney Disease Epidemiology Collaboration; CRP, C-reactive Protein; LVEDd, left ventricular end diastolic diameter; LVEDV, left ventricular end diastolic volume; EF, ejection fraction; RAAS, renin-angiotensin-aldosterone system; SD, Standard deviation.

Chi-square test for categorical variables; Student's t-test for normally distributed continuous variables and Mann-Whitney test and for non-normally distributed continuous variables.

### MINOCA Versus Obstructive Coronary Artery Disease: Secondary Prevention Medical Therapy

At discharge, 75.9% patients with MINOCA were treated with a single antiplatelet drug (aspirin or P2Y12 inhibitor) and only 42.1% with dual antiplatelet therapy (DAPT), compared with 94.6% and 81.8% ob-CAD patients. Similarly, the percentage of MINOCA patients treated with β-blockers, RAAS inhibitor and statins was lower compared with ob-CAD (75.5, 64.7, and 63.9% vs 89.6, 80.3, and 83%, respectively) (**Table 1**). Secondary prevention medical therapy was prescribed in a relatively low proportion of non-ob CAD patients, with coronary plaques (range 63.6–90.9%), and of NCA patients (range 51.5–86.4%) (**Table 3**).

### MINOCA Prognosis

During an average follow up of 19.35 ± 10.6 months), all-cause death occurred in 11 (12.5%) patients, re-MI in 4 (4.5%) patients, and MACE in 15 (17%). No ischemic stroke was observed. Interestingly, all-cause death and recurrent MI were more frequent among patients with NCA than among patients with ob-CAD (15.2 and 6.1% vs 4.5 and 0%, respectively), although this difference was not statistically significant.

**Figure 2:**
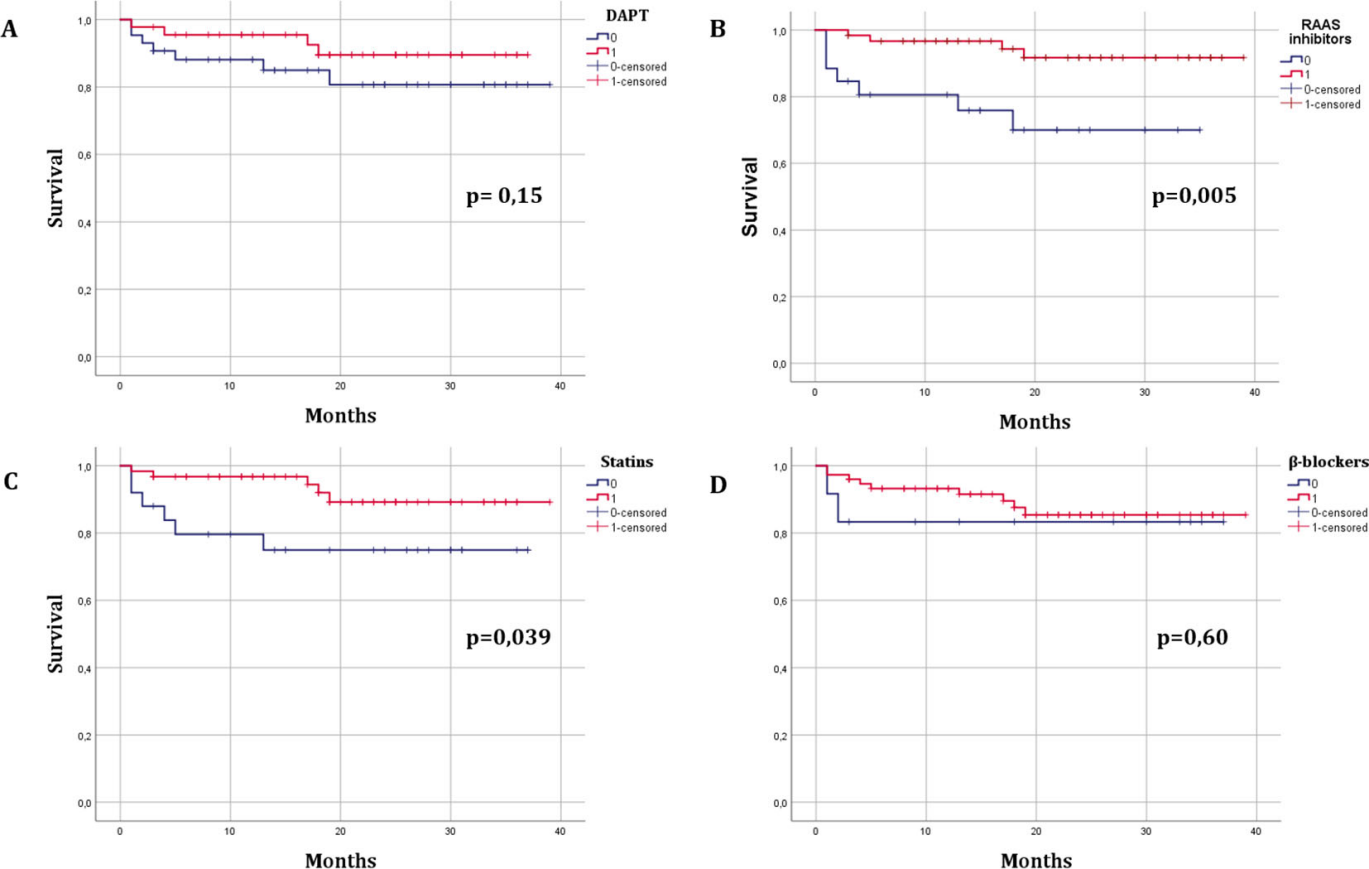
Kaplan-Meier survival curves in MINOCA patients according to treatment with secondary prevention drugs. **(A)**, DAPT; **(B)**, RAAS inhibitors (angiotensin-converting enzyme inhibitors (ACEI) or angiotensin receptor blockers (ARB); **(C)**, statins; **(D)**, B-blockers.

Concerning medications, RAAS inhibitors and statins were the only treatments significantly associated with longer survival in a Kaplan-Meier analysis (**Figure 2**). In univariate Cox regression analyses, a reduced risk of all-cause mortality and MACE was found in patients using RAAS inhibitors (**Table 4**). In a multiple Cox regression analysis including RAAS inhibitors, statins, single antiplatelet therapy (aspirin or P2Y12 inhibitors), DAPT, β-blockers in the model with a forward stepwise procedure, none of these drugs except RAAS inhibitors was associated with lower mortality or with MACE. In contrast, no significant benefit was associated with the use of β-blockers, DAPT or single antiplatelet therapy SAPT (both aspirin or P2Y12 inhibitor).

**Table 4:** Secondary prevention therapy and outcomes.

TOTAL N = 88	MACE HR (95% CI)	Death HR (95% CI)	Re-MI HR (95% CI)
ASA	0.80 (0.23–2.85)	0.93 (0.20–4.33)	0.56 (0.06–5.43)
P2Y12 Inhibitor	0.82 (0.30–2.27)	0.85 (0.26–2.78)	0.73 (0.10–5.19)
DAPT	0.42 (0.14–1.24)	0.48 (0.14–1.64)	0.28 (0.03–2.73)
B-blockers	0.43 (0.14–1.35)	0.67 (0.14–3.09)	0.17 (0.02–1.22)
RAAS Inhibitors	0.29 (0.10–0.81)	0.20 (0.06–0.70)	0.78 (0.08–7.99)
Statins	0.44 (0.16–1.22)	0.31 (0.09–1.01)	1.26 (0.13–12.15)

The associations, HR (95% CI), between treatments and outcomes. CI, confidence interval; HR, hazard ratio; DAPT, dual antiplatelet therapy; RAAS, renin-angiotensin-aldosterone system; MACE, major adverse cardiac events; re-MI, acute myocardial infarction.

## Discussion

This is the first prospective study to evaluate the association between secondary prevention drugs and mid-term outcomes in a selected cohort of patients with MINOCA. Among 88 consecutive MINOCA patients admitted between 2016 and 2018 and followed for an average of 19 months, we found a significantly lower risk of MACE and all-cause of mortality in patients treated with RAAS inhibitors.

### MINOCA: Epidemiology and Prognosis

Nowadays with the common use of coronary angiography in MI, clinicians are regularly confronted with MINOCA which represents a puzzling diagnosis, including heterogeneous patients with many potential etiologies that need to be investigated. In the literature, the prevalence of MINOCA is estimated around 6–8% among patients diagnosed with MI.(5) The rate of MINOCA depends largely on the proportion of patients with MI who performed CAG and on hs-cTn assays used.

In our study the prevalence of MINOCA is around 12%; a possible reason for this discrepancy is that 75% of our patients with acute MI underwent CAG. Indeed, not all patients presenting without ST-segment elevation acute coronary syndromes (ACS) undergo coronary angiography, which may lead to a selection bias (De Ferrari et al., 2014). In fact, if coronary angiograms were increasingly performed in patients with acute myocardial infarction, a higher number of non-obstructive CAD, would be detected.

MINOCA long-term prognosis is not event-free, with an approximate 9.2% rate of MACE per year (Baron et al., 2016; Pizzi et al., 2016; Choo et al., 2019). Interestingly, Kang et al. confirmed that 12-month major adverse cardiac events (death and myocardial infarction) in patients with MINOCA were comparable to those of patients with MI associated with single- or double-vessel coronary artery disease (Kang et al., 2011). Our data regarding MACE are consistent with the literature: we observed about 17% MACE events during the follow-up, which underscores the importance of secondary prevention medical therapy.

### Secondary Medical Therapy Prevention: Under-Prescribed or Not Targeted?

Many clinical studies suggested that MINOCA is an undertreated population; (Ramanath et al., 2010; De Ferrari et al., 2011; Pizzi et al., 2016) however no randomized clinical trial carried out in this population showed that secondary prevention drugs improve the prognosis of these patients.

Consequently, discharging MINOCA patients is challenging for physicians due to the lack of guidelines regarding optimal treatment. Thus, in the absence of a clear alternative, clinicians tend to prescribe atherosclerotic prevention medication. This treatment choice is based on clinical trials that enrolled mostly patients with coronaries obstruction and cannot be extended to MINOCA patients, which represent a different population. Moreover, most drugs used for secondary prevention in patients with MI and obstructive CAD are aimed at counteracting atherosclerotic progression, that constitutes a minor problem in MINOCA in which atherosclerotic plaque disruption is only one of the several related underlying etiologies.

Thus, the crucial issue is whether the prognosis of these patients is not event-free because they are under-treated or because the prescribed drugs are not targeted for this category of patients. Probably, both previous statements are true; in fact, our study confirmed that RAAS inhibitors were the only drug significantly associated with lower all-cause mortality and MACE; in contrast, β-blockers and anti-platelet agents had apparently no effects on outcomes.

The long- term cardiovascular protection conferred by angiotensin-converting enzyme inhibitor-mediated was strongly supported by the results of the Heart Outcomes Prevention Evaluation (HOPE) trial, indicating a significant relative risk reduction in the combined endpoint of cardiovascular death, MI and stroke or cardiac arrest (Yusuf et al., 2000). ESC guidelines recommend the use of RAAS-blockers for effective protection of patients with and without ST-segment elevation MI (Roffi et al., 2016; Ibanez et al., 2018).

The beneficial effects of RAAS inhibitors were already demonstrated in patients with non-obstructive coronary artery disease, however these studies had some limitations such as the definition of MINOCA and inclusion of patients with non-ischemic troponin elevation cause.(9,10,11) Our study confirms that RAAS inhibitors are beneficial in selected patients who meet the 2016 ESC MINOCA Position Paper criteria (Agewall et al., 2017).

The RAAS inhibitors block the ACE-mediated formation of angiotensin (Ag) II (ACE-I) and the linkage of Ag II to receptor type 1 (AT1) (ARBs), in the circulation and peripheral tissues, producing blood pressure lowering, sympathetic inhibition, anti-atherosclerotic, and anti-thrombotic effects(10). In fact, RAAS inhibitors reduce macrophage accumulation, increase bradykinin bioavailability, improving non-endothelial and endothelial dependent coronary microvascular function (Pizzi et al., 2004; Artom et al., 2014). Additionally, they exhibit positive anti-fibrotic effects on the myocardium, by inhibiting macrophage-related inflammation, reducing oxidative stress and transforming growth factor beta (TGF-B) synthesis which is associated with cardiac fibroblast proliferation, collagen deposition, scar formation and cardiac fibrosis (Sun et al., 1998; Tzanidis et al., 2001; Lu et al., 2004).

The “pleiotropic” effects of RAAS inhibitors on the cardiovascular system and their various possible pathways support their benefit for MINOCA patients, a heterogeneous population with different etiology.

As regards statins, survival analysis indicates that they are associated with mortality reduction. The plausible mechanism explaining this effect is the slowdown of the progress of atherosclerotic process and plaque stabilization; (Ambrose et al., 1988) this is especially true in MINOCA patients with mild CAD because plaque disruption can also occur in non-significant plaques. In addition, statins might be beneficial in MINOCA patients through their protective effects on endothelial function (Calabrò and Yeh, 2005).

Although findings from the literature indicate a significant risk reduction in patients with obstructive CAD treated with β-blockers and anti-platelets therapy, no benefits were proved in MINOCA patients. Probably, the many etiopathogenic mechanisms in the absence of athero-thrombotic phenomena associated with complicated plaque and coronary stents support the lack of benefit in antiplatelet prescription. The PROSPECT study demonstrated that ACS occurs from atheroma with definite histopathologic characteristics that are not necessarily dependent on the degree of angiographic stenosis (Stone et al., 2011; Rossini et al., 2013; Crea and Libby, 2017). Indeed, in patients with non-obstructive CAD, activated leukocytes, endogenous fibrinolysis, vasospasm, and embolism are possible mechanisms of acute ischemia without plaque disruption.

### Study Limitations

First, although the prevalence of our population is consistent with that observed in the literature, the number of patients with non-obstructive CAD is relatively small; thus the study is not adequately powered to detect the effect of secondary prevention drugs. Thus, our results should be considered preliminary and warrant confirmation in larger prospective samples. Second, no information was available on the staging and duration of comorbid diseases (i.e.: hypertension, diabetes), and on medication use before hospitalization, which prevents a more detailed investigation on the impact of each comorbidity on outcomes.

Third, angiographic data were retrieved from clinical records and were not adjudicated by a core laboratory, having some degree of between-person variability.

Fourth, no intracoronary investigation other than coronary angiography was performed. Fractional flow reserve or intravascular imaging techniques, like intravascular ultrasound or optical coherence tomography, would likely increase atherosclerotic plaque detection, not visible on CAG (Montone et al., 2018; Rahman et al., 2019). Nowadays, fractional flow reserve (FFR) is used to determine the hemodynamic relevance of coronary artery stenosis. The FAMOUS-NSTEMI trial demonstrated that about 7% of coronary lesions with a stenosis severity of 30–49% in NSTEMI patients exhibited a functional significant (FFR ≤ 0.80) stenosis (Layland et al., 2015). In our study we did not evaluate FFR; however, in our cohort only one third of non-obstructive MINOCA patients presented coronary stenosis severity between 30 and 49%. However, this study reflects the everyday clinical practice in the vast majority of experienced coronary angiography laboratories worldwide, where the use of such techniques is limited due to cost, procedure time and contrast load. Additionally, this “non-atherosclerotic” approach is incomplete because coronary spasm and thrombosis may occur also in the absence of atherosclerosis and it may be an “innocent bystander” in myocardial injury.

## Conclusion

Our data support that treatment with RAAS inhibitor provides mid-term beneficial effects on outcomes in patients with MINOCA; in contrast, we did not find an effect of dual antiplatelet, β-blocker and statin therapy. Our results should be interpreted with caution because the study is under-powered to detect drug effects in a population with a small event rate.

Multicenter prospective adequately powered studies targeting this specific population and the potential benefit of guideline-recommended therapies are warranted.

## Data Availability Statement

All datasets generated for this study are included in the article/supplementary material.

## Ethics Statement

The studies involving human participants were reviewed and approved by Acute Myocardial Infarction; Prognostic and Therapeutic Evaluation' (ClinicalTrials.gov Identifier: NCT03883711). The patients/participants provided their written informed consent to participate in this study.

## Author Contributions

PP, LB, and CP contributed conception and design of the study. GS, IM, ED'A, ST, AS, AR, LB, FA, and FD organized the database. LB and PR performed the statistical analysis. PP wrote the first draft of the manuscript. PP, CP, and NT wrote sections of the manuscript. AM and NG revised the article. All authors contributed to manuscript revision, read and approved the submitted version.

## Conflict of Interest

The authors declare that the research was conducted in the absence of any commercial or financial relationships that could be construed as a potential conflict of interest.
